# Real-time jellyfish classification and detection algorithm based on improved YOLOv4-tiny and improved underwater image enhancement algorithm

**DOI:** 10.1038/s41598-023-39851-7

**Published:** 2023-08-10

**Authors:** Meijing Gao, Shiyu Li, Kunda Wang, Yang Bai, Yan Ding, Bozhi Zhang, Ning Guan, Ping Wang

**Affiliations:** 1 School of Integrated Circuits and Electronics, Beijing Institute of Technology, Beijing, 100081 China; 2 The Key Laboratory for Special Fiber and Fiber Sensor of Hebei Province, College of Information Science and Engineering, Yanshan University, Qinhuangdao, 066004 Hebei China; 3 The Key Laboratory of Dynamics and Control of Flight Vehicle, Ministry of Education, School of Aerospace Engineering, Beijing Institute of Technology, Beijing, 100081 China

**Keywords:** Optical imaging, Marine biology, Computer science

## Abstract

The outbreak of jellyfish blooms poses a serious threat to human life and marine ecology. Therefore, jellyfish detection techniques have earned great interest. This paper investigates the jellyfish detection and classification algorithm based on optical images and deep learning theory. Firstly, we create a dataset comprising 11,926 images. A MSRCR underwater image enhancement algorithm with fusion is proposed. Finally, an improved YOLOv4-tiny algorithm is proposed by incorporating a CBMA module and optimizing the training method. The results demonstrate that the detection accuracy of the improved algorithm can reach 95.01%, the detection speed is 223FPS, both of which are better than the compared algorithms such as YOLOV4. In summary, our method can accurately and quickly detect jellyfish. The research in this paper lays the foundation for the development of an underwater jellyfish real-time monitoring system.

## Introduction

With the rapid development of deep-sea exploration technology, people pay increasing attention to the exploration and utilization of marine resources, which is crucial for acquiring, collecting, and rationally using navigational information^1^.

The Bohai sea, situated in eastern China, is rich in marine resources. However, the local economy and ecosystem have been significantly impacted by periodic catastrophes caused by marine creatures such as red tide, green algae, and jellyfish floods^2,3^. Among these calamities, jellyfish blooms have garnered international attention as a prominent maritime ecological concern. Nonetheless, real-time jellyfish detection remains challenging due to the complex marine environment and the immature related detection technologies^4^.

In the past, oceanographers typically fished jellyfish by collecting water samples or using simple trawl technique. Subsequently, these jellyfish specimens were brought to laboratories for manual identification. While this approach allows for effective research and statistical analysis of jellyfish species, it requires significant human and material resources. In an attempt to address these challenges, some scholars have utilized mathematical modeling and biological detection techniques for jellyfish detection. However, these methods possess notable limitations and are unable to provide real-time detection^5–7^. Furthermore, they can inadvertently harm living beings, making it challenging to satisfy the demands of real-time detection and identification^8^.

The advancement of underwater optical and acoustic imaging has led to a high degree of jellyfish monitoring. Sonar and optical imaging are relatively mature among the various jellyfish detection technologies^9,10^.

Sonar imaging monitors jellyfish by transmitting and receiving sonar signals, but the resulting images have low-resolution and cannot distinguish between species^11^. On the other hand, Optical imaging technology has been widely used in jellyfish monitoring compared to sonar imaging. This is primarily due to its high image resolution, non-contact, real-time imaging, and species identification advantages. In addition, as jellyfish are slow-moving creatures, obtaining clear images through optical equipment is relatively straightforward.

An innovative target monitoring technique is the Convolutional Neural Network (CNN), which has progressively become a novel monitoring approach to study marine life. The CNN-based target identification algorithm is primarily split into two groups: regression-based algorithms like YOLOv4-tiny and Faster R-CNN, which are based on region recommendations.

The research first employs the YOLOv4-tiny since the Faster R-CNN has the issue of low real-time. However, when the YOLOv4-tiny algorithm is used, there is a problem with poor accuracy.

The organization of the rest of this article is as follows. "Related research and contributions" section introduce the related works on jellyfish detection and their limitations. "Dataset preparation and preprocessing" section presents the establishment of the dataset and underwater image enhancement algorithm. Section "Improved YOLOv4-Tiny Jellyfish Detection Algorithm" describes the jellyfish classification method based on the improved YOLOv4-tiny algorithm. "Experiment and result analysis" section shows experimental results to validate the effectiveness and robustness. The conclusion is provided in "Conclusions" section.

## Related research and contributions

This section introduces the existing work related to the detection and monitoring of jellyfish and then presents the paper’s main contributions.

### Related research literature

Underwater image processing is a necessary means to improve detection accuracy. Therefore, in this paper, we first introduce recent research on underwater image processing. In 2018, Lu et al. proposed the guided image filtering for contrast enhancement method to improve the quality of underwater images. However, the guided filter they used could only be applied to grayscale images, and therefore performed poorly in color restoration^12^.

In 2021, Liu studied underwater image restoration algorithms based on the dark channel prior method, which improved the restoration effect to some extent. However, due to the large number of parameters, the algorithm's robustness was poor^13^.

In 2022, Li et al. proposed the Dark Channel and MSRCR Algorithm Combined method to achieve underwater image dehazing and enhancement, but the method had poor scene applicability^14^.

In 2022, Zhou et al. proposed an algorithm for automatic color correction of underwater images, which solved the color cast caused by the attenuation difference of different color channels in underwater images and could adapt to various underwater environments^15^.

In 2023, Zhou et al. further proposed the multi-interval sub-histogram perspective equalization method for underwater enhancement, which achieved contrast enhancement of underwater images through adaptive interval partitioning and histogram equalization, with excellent image restoration effects^16^.

Furthermore, we introduce related research on jellyfish detection technology. Due to the harm and research value of jellyfish, researchers have long used various technologies, including acoustic, optical, and remote sensing, to search for jellyfish. In 1994, Davis et al. designed a submarine plankton video recording system. Rich visual information, quick recording, and the capacity to capture in-depth jellyfish movement are all benefits of the technology^17^.

In 2006, Houghton et al. recorded jellyfish movement characteristics and distribution using aerial photography technology. However, this approach could only observe large-sized jellyfish near the sea’s surface^18^.

In 2015, Donghoon Kim et al. developed an autonomous jellyfish detection and cleaning system. At the same time, the team also proposed a jellyfish detection algorithm based on drone photography. However, it cannot identify jellyfish^19^.

In 2016, Seonghun Kim et al. investigated jellyfish’s spatial and vertical distribution by acoustic and optical methods, but this method had limitations in monitoring tiny jellyfish^20^. Hangeun Kim et al. put forward a drone detection system for jellyfish. The design captured the movements of jellyfish on the sea surface and recognized them through deep learning. However, it was limited to the *Aurelia aurita*^21^.

In 2017, Jungmo Koo and colleagues developed a system seeking out jellyfish distribution by crewless aerial vehicles. They employed a deep neural network that demonstrated high precision and fast speed in accurately identifying jellyfish. Nonetheless, it is noteworthy that this approach can solely discriminate a singular jellyfish species^22^. Martin-Abadal et al. used neural network to design a Jelly monitoring system for the automatic detection and quantification of various jellyfish types, as well as enabling long-term monitoring of their presence. However, the system's applicability is constrained^23^.

In 2018, French et al. implemented underwater imaging technology and neural network to monitor and classify jellyfish. The accuracy of classification reached up to 90%, suggesting that the system can serve as an effective tool for predicting jellyfish outbreaks. However, the system is limited to detect individual jellyfish outbreaks.

In 2020, a novel technique for the automated detection and quantification of jellyfish was developed by Martin Vodopiveca et al. This approach enables the continuous monitoring of jellyfish, while simultaneously assessing the accuracy of manual counting^24^. Through the use of optical imaging and automated image analysis, the algorithm demonstrates the feasibility of identifying jellyfish. However, the current implementation remains limited to offline recognition and is not yet capable of real-time monitoring.

In 2021, Chang Qiuyue et al. of Yanshan University proposed an improved YOLOv3 algorithm, which can achieve real-time detection and identify seven jellyfish species. But its speed and accuracy need to be improved^25^.

In the past, although a series of studies have been carried out on jellyfish detection using acoustics and optics combined with deep learning theory, the research on jellyfish detection is still in the primary stage. So, further study and improvement are needed to improve detection accuracy, speed, and species identification.

### Contributions

The main contributions of this paper are as follows:

1. A dataset containing seven species of jellyfish and fish is established in "Dataset preparation and preprocessing" section, including *Cyanea purpurea*, *Rhizostoma pulmo*, *Phacellophora camtschatica*, *Agalma okeni*, *Aurelia aurita*, *Phyllorhiza punctata*, *Rhopilema esculentum*, and *fish*, a total of 11,926 images.
2. An underwater image enhancement algorithm is proposed based on Multi-Scale Retinex with Color Restoration (MSRCR) and an underwater image fusion algorithm, to solve severe blurring and color degradation of underwater images.
3. An attention mechanism module is added in the feature extraction network of the YOLOv4-tiny algorithm, to improve the feature extraction ability and strengthen the ability to identify small and occluded targets.
4. Mosaic enhancement, which enhances the data at the network's input when training the network, is added. Meanwhile, label smoothing and cosine annealing learning rate training methods are applied to improve the overall detection effect of the algorithm.

## Dataset preparation and preprocessing

### Dataset preparation

**Figure 1:**
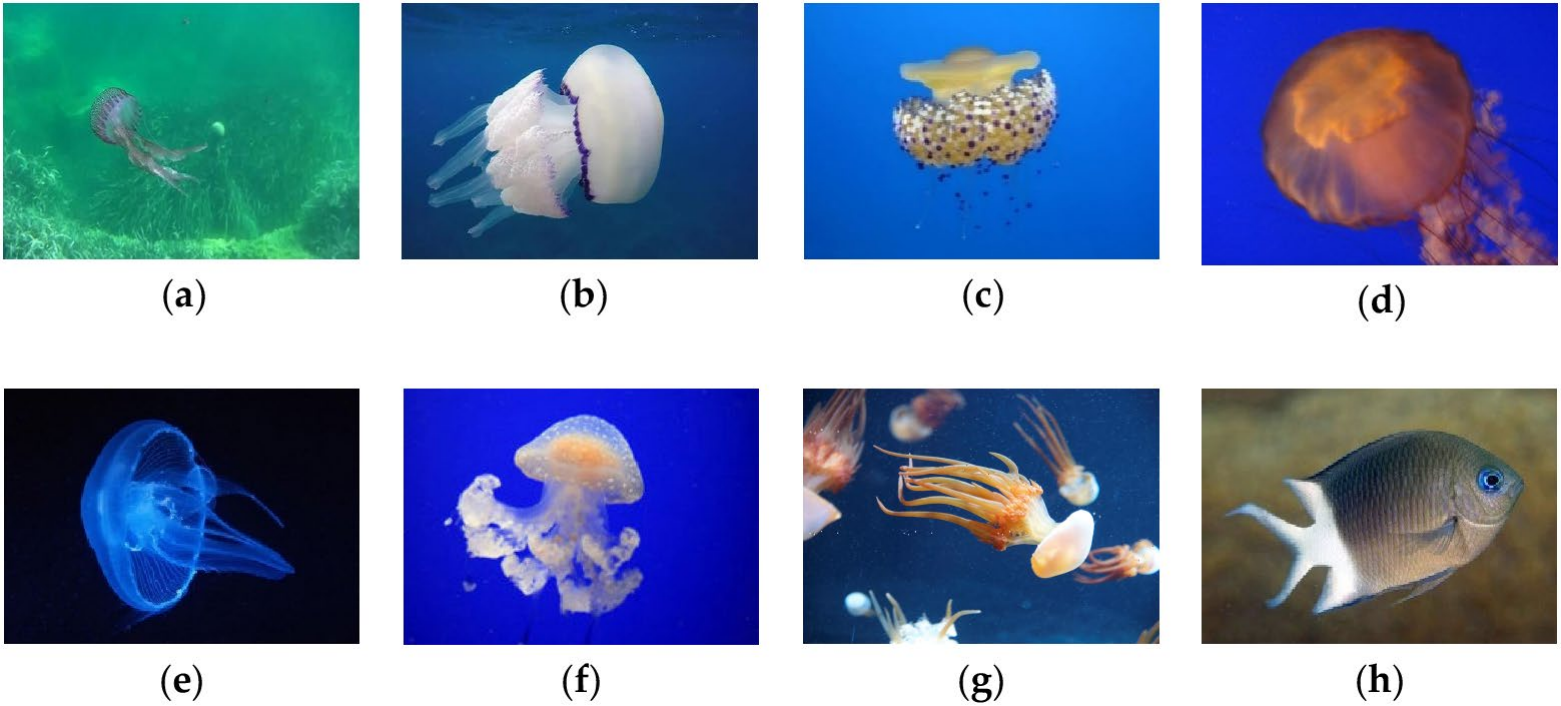
The example of target samples. (**a**) *C. purpurea;* (**b**) *R. pulmo;* (**c**) *P. camtschatica;* (**d**) *A. okeni;* (**e**) *A. aurita;* (**f**) *P. punctata;* (**g**) *R. esculentum*; (**h**)* fish*.

The dataset used in our study comprises jellyfish images obtained through two sources: Crawler technology and our own lab. The jellyfish to be recognized are categorized into eight classes, including disruptor fish and seven jellyfish species, which are *Cyanea purpurea*, *Rhizostoma pulmo*, *Phacellophora camtschatica*, *Agalma okeni*, *Aurelia aurita*, *Phyllorhiza punctata*, *Rhopilema esculentum*, and *fish*. Among them, *C. purpurea*, *R. pulmo*, and *P. camtschatica* are derived from the public dataset by Miguel Martin-Abadal et al.^23^. The online websites were utilized for obtaning the dataset on *A. okeni*, *P. punctata*, the majority of *A. aurita*, and *fish*. Additionally, data on *R. esculentum* and some *A. aurita* were collected in our lab. The dataset consists of a total of 2141 photos. Figure 1 displays the images of various jellyfish.

Supervised data augmentation methods are employed for dataset augmentation. The dataset is divided into the training set, the verification set, and the test set. The distribution of the dataset is shown in Table 1.

**Table 1:** Dataset distribution after augmentation.

Dataset settings	Images number
The training set	9594
The verification set	1067
The test set	1265
Total	11,926

### Underwater image preprocessing

**Figure 2:**
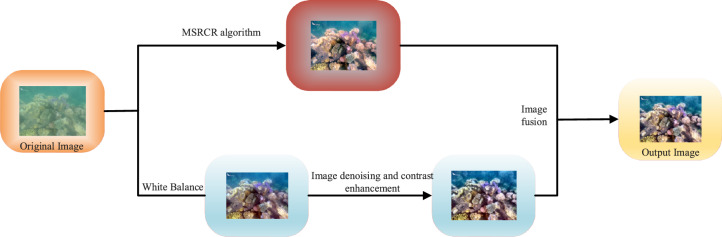
Flow chart of improved underwater image enhancement algorithm.

First, we use MSRCR combined with an underwater image fusion method to address the issue of color deterioration and blurring of underwater images acquired by optical equipment. The MSRCR, known for its ability to enhance color in input images^26^, is employed as the initial technique in our new algorithm. The second method focus on image denoising and contrast enhancement^27–29^. Subsequently, the output images generated by these two methods are merged using an underwater fusion algorithm, resulting in a final image with vibrant color, sharp contrast, and distinct texture^30^. For a more comprehensive understanding of the process, please refer to Fig. 2, which illustrates the detailed steps of this new algorithm.

**Figure 3:**
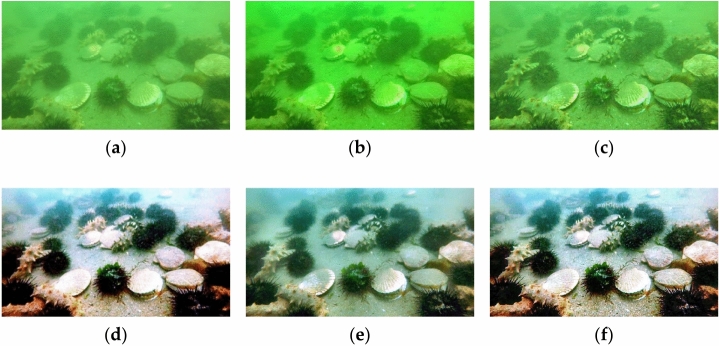
Effects of five algorithms. (**a**) Original image; (**b**) dark channel prior defogging; (**c**) contrast enhancement; (**d**) MSRCR; (**e**) underwater fusion; (**f**) improved algorithm.

In our study, we employ five different algorithms to process the original image. These algorithms include the dark channel prior defogging^13^, contrast enhancement proposed by Lu et al.^12^, MSRCR^26^, underwater image fusion, and our suggested improved underwater image enhancement algorithm. The resulting images are subsequently evaluated using four evaluation parameters: Entropy^29^, UCIQE^31^, UIQM^32^ and EOG^33^. Figure 3 visually presents the effects achieved by applying five algorithms. The evaluation results are given in Table 2.

**Table 2:** Average evaluation results.

Parameters	Entropy	UCIQE	UIQM	EOG
Dark channel prior defogging	7.6760	0.3966	0.0056	3.8187
Contrast enhancement	7.6034	0.3777	− 0.2182	4.6496
MSRCR	7.5732	0.4783	0.4655	8.9924
Underwater fusion	7.8525	0.4459	0.0813	6.0049
Improved algorithm	7.8583	0.4791	0.4504	9.0601

**Figure 4:**
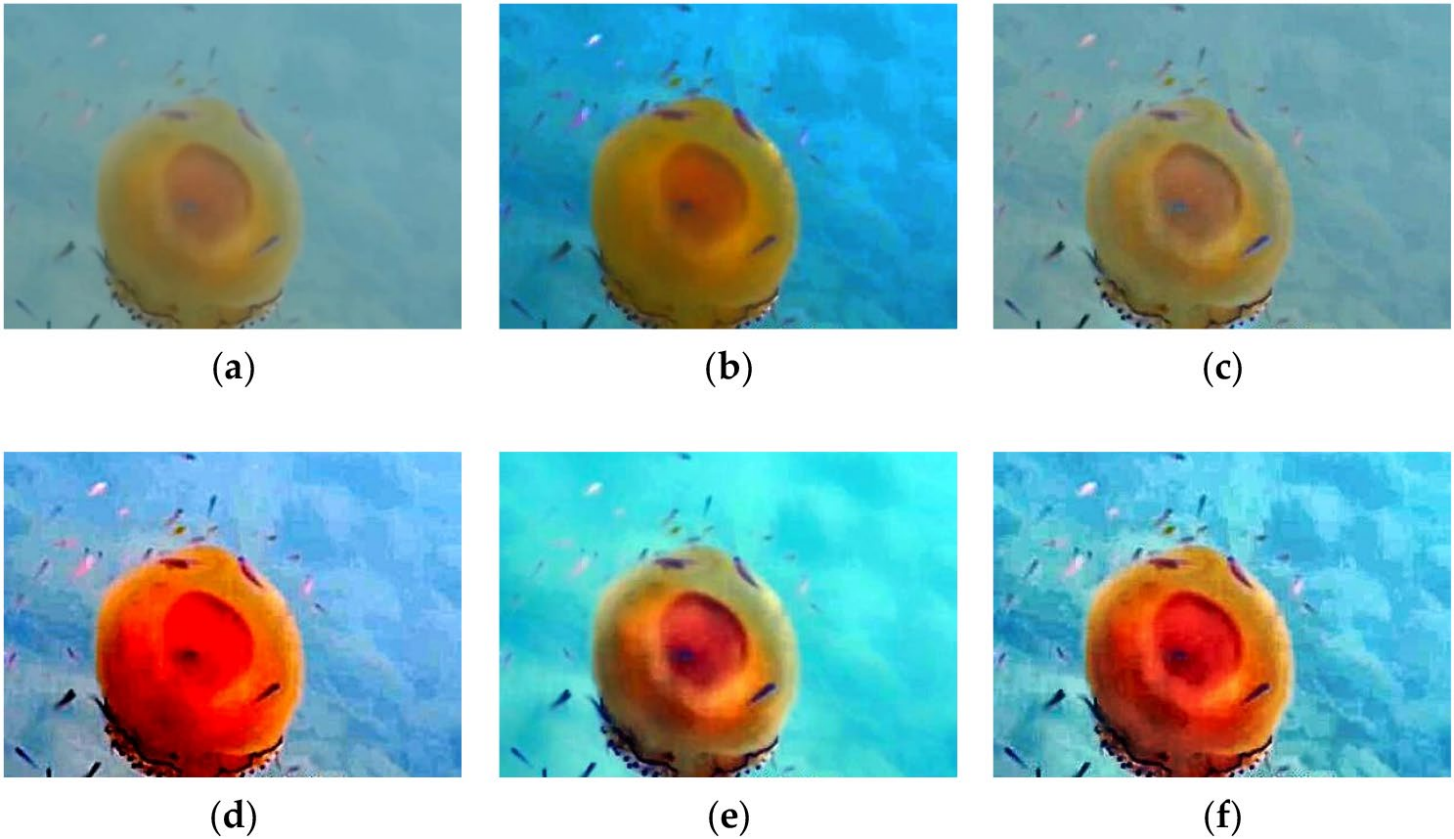
Results of five algorithms. (**a**) Original image; (**b**) dark channel prior defogging; (**c**) contrast enhancement; (**d**) MSRCR; (**e**) Underwater fusion; (**f**) improved algorithm.

It can be seen from Table 2 that the Entropy, UCIQE, and EOG reach their maximum when the improved algorithm is applied. Moreover, the improved algorithm can also fulfill the demands of exhibiting the target items and increasing the color of underwater images. Above all, the improved algorithm presented in this section demonstrates excellent performance and could be applied to enhance the optical images utilized in the jellyfish detection system. Figure 4 shows the results processed by five algorithms.

Some conclusions can be derived from Fig. 4a–f: The dark channel prior defogging algorithm produces limited color recovery and discernible changes in image texture details. The contrast enhancement method primarily focuses on recovering image texture details, with subpar color recovery. The MSRCR algorithm performs well in image color recovery, but compromises the texture information. While the contrast of the picture backdrop is somewhat increased, the color recovery impact of the fusion algorithm is inferior to that of MSRCR. The new algorithm, on the other hand, produces the most satisfactory overall result. It achieves a mild yet effective color recovery, exact image texture details, and high contrast.

## Improved YOLOv4-Tiny Jellyfish Detection Algorithm

The YOLO algorithms have gained widespread popularity in target detection applications. Among them, the YOLOv4-tiny algorithm has fast detection speed, relatively high detection accuracy, a simple network model, and low hardware needs^34–36^. The YOLOv4-tiny has a significantly faster recognition speed than YOLOv4, but its accuracy has declined^37^. Therefore, this work will adopt two ways to enhance the YOLOv4-tiny algorithm's accuracy and make it compliant with the criteria of jellyfish detection accuracy and speed. Specific improvements are: (1) Add the attention mechanism module to improve the feature extraction ability of the network and strengthen its recognition of obscured and tiny targets. (2) The mosaic data enhancement is used at the network's input when training the network. To enhance the overall detection impact, two training techniques are simultaneously introduced: label smoothing and the cosine annealing learning rate.

### Add CBAM

CBAM is an attention mechanism that combines space and channel^38^. Compared with the mechanism that only focuses on one channel attention mechanism, the hybrid attention mechanism can achieve better results. Therefore, the hybrid attention mechanism is introduced to make the neural network concentrate more on the target areas that contain essential information and suppress irrelevant information, thereby improving accuracy.

**Figure 5:**
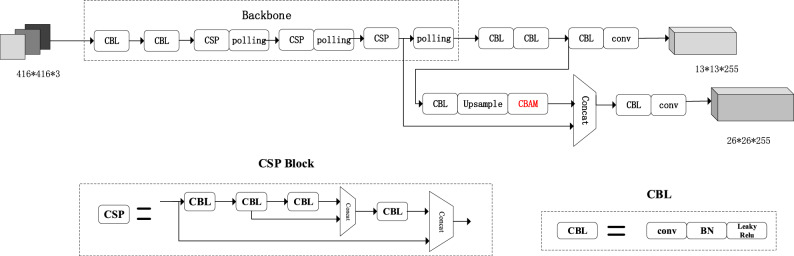
Network structure for adding the CBAM.

The YOLOv4-tiny obtains feature information through the neural network, and there is no feature extraction step. As a result, it is simple to overlook tiny targets and obscured objects. In the paper, the CBAM is added after upsampling. The attention mechanism can weight the feature data of the target objects with dynamic weight coefficients, thus improving the network's ability to pay attention to the target objects, solving the problem of small targets and occluded objects being ignored. Figure 5 depicts the network topology for adding the CBAM.

### Improvements in training methods

To improve the detection accuracy further, we will introduce the mosaic data enhancement, cosine annealing learning rate and label smoothing in this section.

Mosaic is a form of data enhancement used before model training. Its purpose is to merge four random images into a single new image, thereby enriching the background of the detection target. This process enhances the variety and informational content of the input images, while also reducing overfitting. The steps involved in the mosaic data enhancement are as follows:

1. Read four random images;
2. Crop, zoom, flip, and color gamut changes for four images, respectively;
3. The images from the second step are stitched to obtain images in the specified size range.

**Figure 6:**
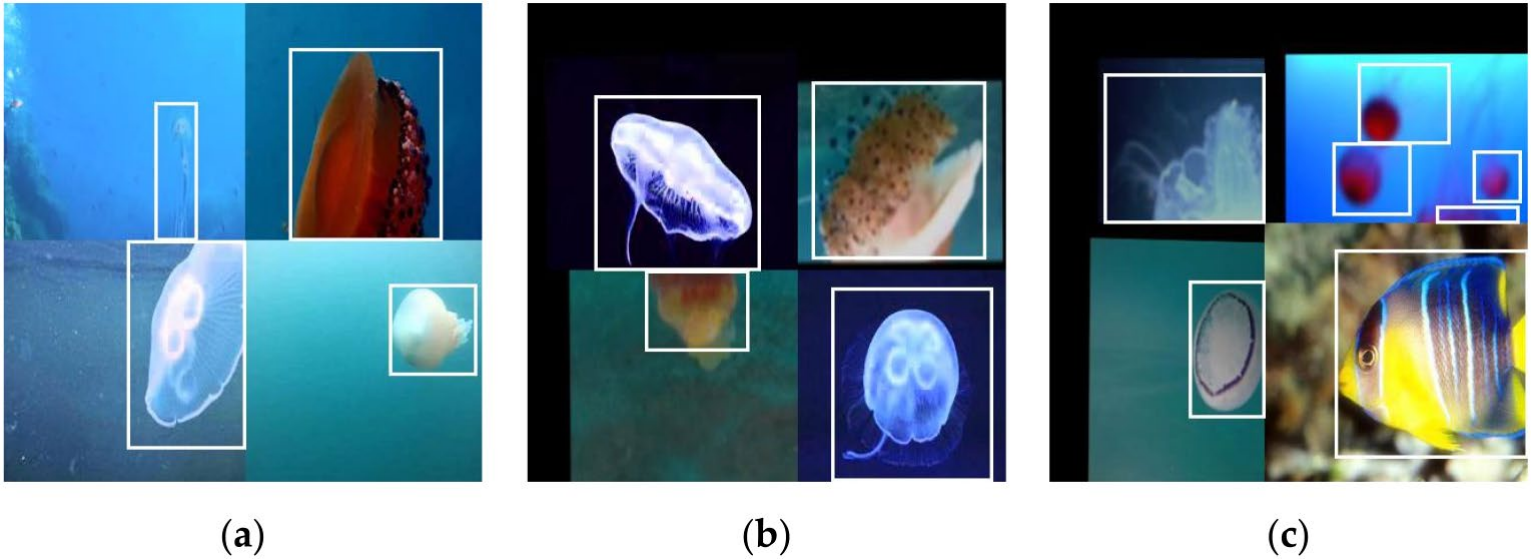
Mosaic enhanced images.

The mosaic enhanced images are shown in Fig. 6.

Cosine annealing learning rate can reduces the learning rate using a cosine function. Initially, the model enters the training state with a gradually decreasing function value. This faster decrement leads to accelerated convergence of the learning rate. Subsequently, the learning rate gradually decreases again to prevent overshooting the optimal point. This approach often yields favorable results.

The majority of jellyfish have long tentacles and umbrella-shaped heads, with striking similarities. Because of this, manual labeling will inevitably result in mistakes that will impact on the final predictions. Label smoothing prevents over-trust by assuming that labels may be incorrect during training. In this chapter, label smoothing is introduced to improve accuracy. The smoothing coefficient is 0.01.

### Comprehensively improved algorithm

**Figure 7:**
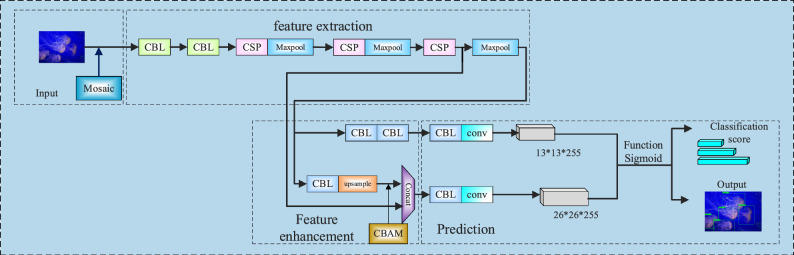
Structure of comprehensively improved algorithm.

Combining the improved network and training method, a comprehensively improved algorithm is obtained, and the structure is depicted in Fig. 7.

## Experiment and result analysis

### Experimental process

1. Experimental method
  Seven tests are carried out to verify the improved algorithm and training method. The seven groups of experiments are as follows: (1) the original YOLOv4 algorithm; (2) the original YOLOv4-tiny algorithm; (3) the improved YOLOv4-tiny network adding CBMA; (4) the improved network and mosaic enhancement; (5) the improved network and cosine annealing learning Rate; (6) the improved network and label smoothing; (7) Comprehensively improved algorithm (including the improved network, mosaic enhancement, cosine annealing learning rate, and label smoothing).
  The network is trained separately with the original data and the enhanced data in "Dataset preparation" section. The train, valid and test sets is set as Table 1.
2. Parameter settings
  The hyperparameter settings are shown in Table 3.

**Table 3:** Hyperparameter settings.

Hyperparameter	YOLOv4	YOLOv4-tiny	Improved algorithm
Learning rate	1e−4	1e−4	Cosine annealing learning rate
Loss function	YOLOloss		
Optimizer	Adam		
Batch size	4	4	16
Epoch	Train to model convergence

### Algorithm comparison

To demonstrate the effectiveness and superiority of our proposed method, we compared it with several classical and state-of-the-art methods, including YOLOV4, YOLOV5, YOLOV6, YOLOV7, YOLOV8, and our methods. In order to compare the different algorithms more effectively and intuitively, we compared their complexity and accuracy, and the results are shown in Table 4. Layers, parameter quantity, and FPS reflect the complexity of the algorithm. Lower values of layers and parameter quantity indicate simpler architecture with fewer generated parameters and lower complexity, while higher FPS indicates faster processing speed. mAP and F1 reflect the accuracy of the algorithm, with higher values indicating better performance. As shown in Table 4, our proposed algorithm maintains a lightweight structure while achieving high detection performance.

**Table 4:** Comparison and evaluation results of algorithms.

Metrics	YOLOV3-tiny	YOLOV5	YOLOV6	YOLOV7	YOLOV8	Ours
Layers	63	193	142	314	168	38
Parameter quantity	12,131,776	2,504,504	4,234,536	36,519,530	3,007,208	5,925,313
FPS	160	142	143	103	125	162
mAP	90.1	88.2	88.3	93.1	89.4	94.6
F1	0.86	0.74	0.75	0.89	0.80	0.90

**Figure 8:**
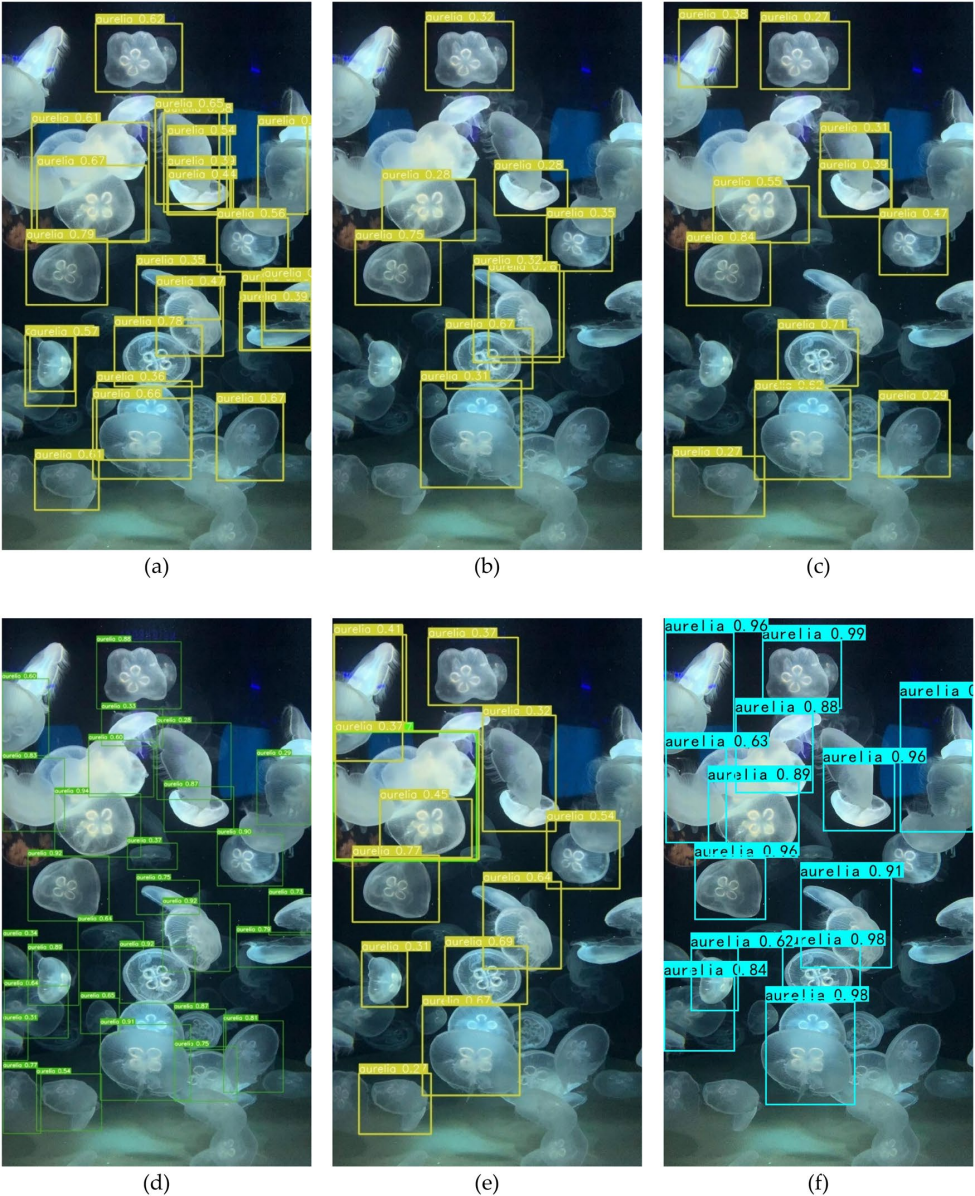
Results of Algorithms for aurelia. (**a**) YOLOv3-tiny; (**b**) YOLOv5; (**c**) YOLOv6; (**d**) YOLOv7; (**e**) YOLOv8; (**f**) Ours.

Figure 8 shows the results of different methods trained on the same dataset in the comparative experiment of jellyfish detection. From the Fig. 8, it can be analyzed that regardless of the method used, both false positives and false negatives occurred in the detection of multiple jellyfish images, which proves that jellyfish detection is a challenging task. Among the detection results, the proposed algorithm has the highest confidence but with significant false negatives. YOLOv7 detected the most jellyfish and maintained a high level of confidence. YOLOv5, YOLOv6, and YOLOv8 did not perform well in jellyfish detection. Therefore, we can consider the YOLOv7 method as a deadline for jellyfish detection, while other methods still need improvement.

### Ablation experiment process

The experimental data are quantitatively analyzed in this part using five evaluation indices, and the findings are as follows:

1. Average precision (AP) value analysis
  Tables 5 and 6 are the results of the above seven experimental methods.

**Table 5:** The AP results of seven algorithms with original dataset.

Jellyfish species	YOLOv4	YOLOv4-tiny	Improved network structure	Improved network structure			Comprehensively improved algorithm
				Mosaic	Cosine annealing learning rate	Label smoothing	
*C. purpurea*	93.21	94.26	95.50	94.05	96.22	96.69	95.68
*R. pulmo*	99.83	98.11	98.13	98.09	98.24	97.97	98.81
*P. camtschatica*	99.88	99.87	99.89	99.83	99.91	99.7	99.79
*A. okeni*	76.16	78.40	79.02	78.39	79.64	81.43	82.01
*A. aurita*	94.88	93.97	94.30	93.90	94.27	94.63	94.79
*P. punctata*	93.36	94.43	94.46	95.21	93.27	93.90	94.21
*fish*	91.76	95.00	93.93	94.96	94.06	91.98	93.29

**Table 6:** The AP results of seven algorithms with enhanced dataset.

Jellyfish species	YOLOv4	YOLOv4-tiny	Improved network structure	Improved network structure			Comprehensively improved algorithm
				Mosaic	Cosine annealing learning rate	Label smoothing	
*C. purpurea*	97.42	94.14	97.00	95.15	98.22	96.36	95.02
*R. pulmo*	96.87	98.21	99.91	99.78	98.98	99.51	99.19
*P. camtschatica*	99.48	100.00	99.95	99.99	99.93	99.77	99.94
*A. okeni*	82.01	76.56	80.95	76.73	84.32	84.38	80.54
*A. aurita*	94.44	92.83	94.80	95.55	95.76	94.67	95.54
*P. punctata*	93.67	89.37	92.62	94.10	94.46	93.78	95.22
*fish*	93.65	92.76	92.82	99.56	89.64	92.15	97.41
  Tables 5 and 6 indicate that the utilization of data enhancement has resulted in improved Average Precision (AP) values for most jellyfish species. In addition, the comprehensively improved algorithm achieves a detection accuracy over 95% for most jellyfish types. The mean average precision (mAP) of the seven algorithms is listed in Table 6.
  It can be seen from Table 7 that, except for the original YOLOv4-tiny algorithm, the mAP of the other six algorithms is higher than the values without data enhancement, proving the effectiveness of data enhancement. Comparing the mAP values obtained by various methods, it can be seen that the mAP of the YOLOv4-tiny algorithm after data enhancement is 1% lower than that of the YOLOv4 algorithm, this indicates a reduction in the feature extraction capability of the YOLOv4 network due to its simplified structure. Table 6 further demonstrates that the improved network structure leads to a 1.59% increase in mAP compared to YOLOv4-tiny and a 0.59% increase compared to YOLOv4, Among the different enhancements, mosaic enhancement produces the most significant effect, surpassing the impact of cosine annealing learning rate and label smoothing.

**Table 7:** The mAP values of seven algorithms.

Dataset	YOLOv4	YOLOv4-tiny	Improved network structure	Improved network structure			Comprehensively improved algorithm
				Mosaic	Cosine annealing learning rate	Label smoothing	
Enhanced	91.93	93.31	93.68	93.55	93.85	93.51	94.12
Original	93.46	92.46	94.05	94.66	94.54	94.35	95.01
  The mAP can reach 95.01% utilizing data enhancement and the improved algorithm, which is 2.55% higher than the original YOLOv4-tiny algorithm, illustrating that the improved algorithm has the highest detection accuracy. The bold data in the table is the mAP value of the comprehensively improved algorithm.
2. FPS analysis
  Table 8 displays the FPS values for the seven algorithms. From Table 8, we can see that the detection speed of the YOLOv4-tiny algorithm can reach 248 FPS, which is nearly five times higher than that of the YOLOv4 algorithm with only 43.9 FPS. When the average accuracy is considered, it is clear that YOLOv4-tiny will sacrifice a small amount of precision to increase detection speed. The detection speed of the comprehensively improved algorithm can reach 223 FPS, which is a little different from the original YOLOv4-tiny algorithm. As a result, the comprehensively improved algorithm improves the detection accuracy while sacrificing a bit of the detection speed.

**Table 8:** The FPS of seven algorithms.

Dataset	YOLOv4	YOLOv4-tiny	Improved network structure	Improved network structure			Comprehensively improved algorithm
				Mosaic	Cosine annealing learning rate	Label smoothing	
Enhanced	42.5	246	224	225	181	226	224
Original	43.9	248	198	208	208	220	223
3. Precision analysis
  Table 9 shows the average precision of each algorithm. Table 9 shows that the accuracy of the comprehensively improved algorithm after the data enhancement can reach 92.56%, which can satisfy the detection criteria.

**Table 9:** The precision of seven algorithms.

Dataset	YOLOv4	YOLOv4-tiny	Improved network structure	Improved network structure			Comprehensively improved algorithm
				Mosaic	Cosine annealing learning rate	Label smoothing	
Enhanced	89.95	89.94	88.44	88.93	89.56	90.55	91.15
Original	95.37	92.28	92.38	94.23	94.54	91.63	92.62
4. Recall analysis
  The average recall of the seven algorithms introduced in this chapter is shown in Table 10. When processing the enhanced dataset, we can see that the recall rate of the comprehensively improved algorithm can reach 89.69%, which is the highest figure among all methods. The bold data in the table are the evaluation parameters obtained by the comprehensively improved algorithm. The mAP and Recall values of the seven methods both before and after data improvement are summarized in Fig. 9.

**Table 10:** The recall value of seven algorithms.

Dataset	YOLOv4	YOLOv4-tiny	Improved network structure	Improved network structure			Comprehensively improved algorithm
				Mosaic	Cosine annealing learning rate	Label smoothing	
Enhanced	89.62	88.67	88.44	88.93	89.56	88.39	88.65
Original	89.51	87.78	88.19	88.80	88.21	89.06	89.69

**Figure 9:**
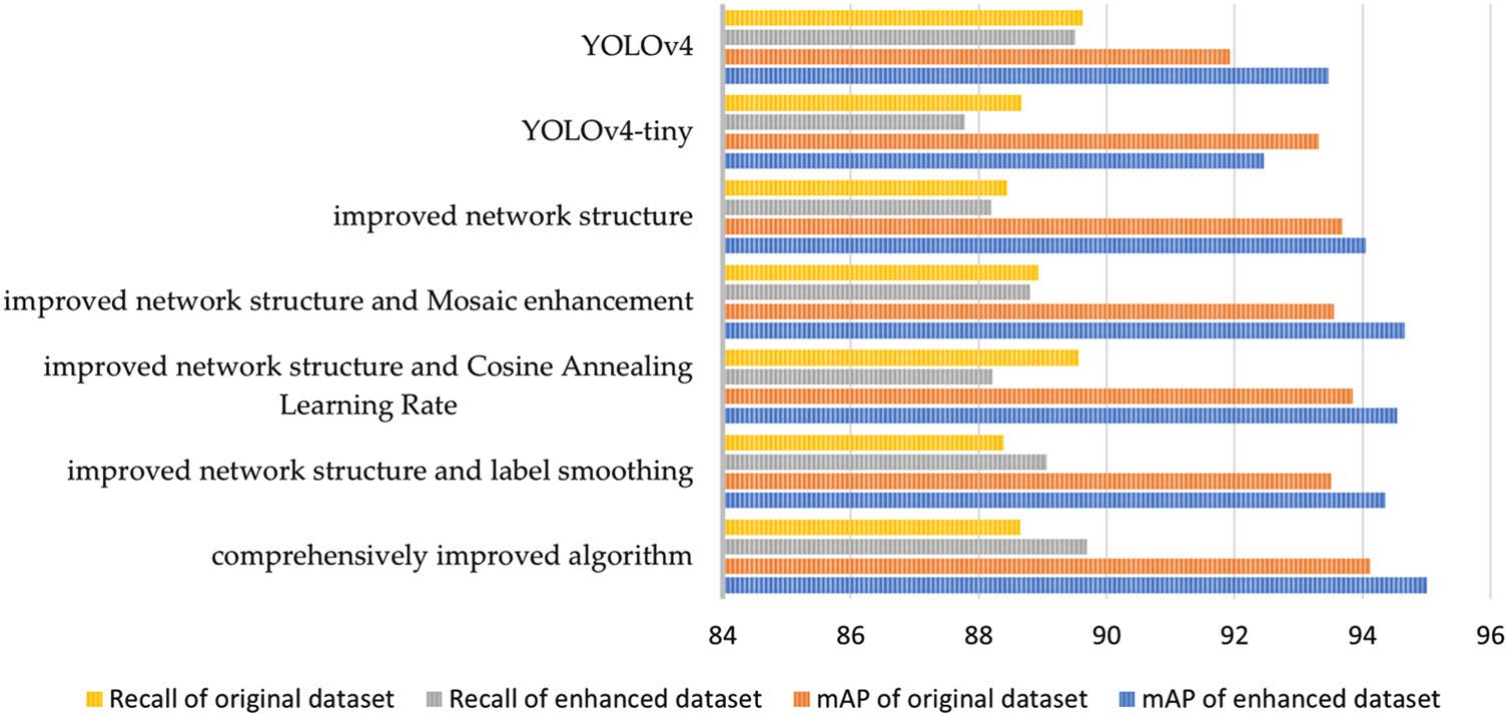
The mAP and recall values of seven algorithms.
5. F1 score analysis
  The comparison of the F1 Score of the seven algorithms is shown in Table 11. The greater the F1 Score, the better the network performance since it indicates how well the model can balance accuracy and recall. It can be seen that the F1 Score of the seven algorithms all distribute around 0.90, and the comprehensively improved algorithm has the most considerable F1 Score.

**Table 11:** The F1 scores of seven algorithms.

Dataset	YOLOv4	YOLOv4-tiny	Improved network structure	Improved network structure			Comprehensively improved algorithm
				Mosaic	Cosine annealing learning rate	Label smoothing	
Enhanced	0.90	0.89	0.90	0.90	0.90	0.90	0.90
Original	0.91	0.90	0.90	0.91	0.92	0.90	0.91
  The seven algorithms are evaluated in this section using the aforementioned objective assessment indices of accuracy, FPS, precision, recall, and F1 score. The results show that YOLOv4-tiny dramatically improves the detection speed compared to the YOLOv4 algorithm while sacrificing a little precision. Its detection speed is six times that of YOLOv4, and other evaluation indicators are almost equal. The detection accuracy of the comprehensively improved algorithm has been improved, and the detection speed can be maintained above 220 FPS, which can meet the demands for rapid testing. The comprehensively improved algorithm has 95.01% accuracy and 223 FPS, slightly less than the improved YOLOv3 method previously proposed in our lab, which had 95.53% accuracy and 52.53 FPS detection speed^39^. The detecting speed has, however, greatly increased and is now four times faster than the prior method. Through the objective evaluation and analysis, it can be seen that the comprehensively improved algorithm has the best overall impact. It quantitatively illustrates the effectiveness of the comprehensively improved algorithm we proposed. Figure 10 shows the image results processed by different methods in the ablation experiment.

**Figure 10:**
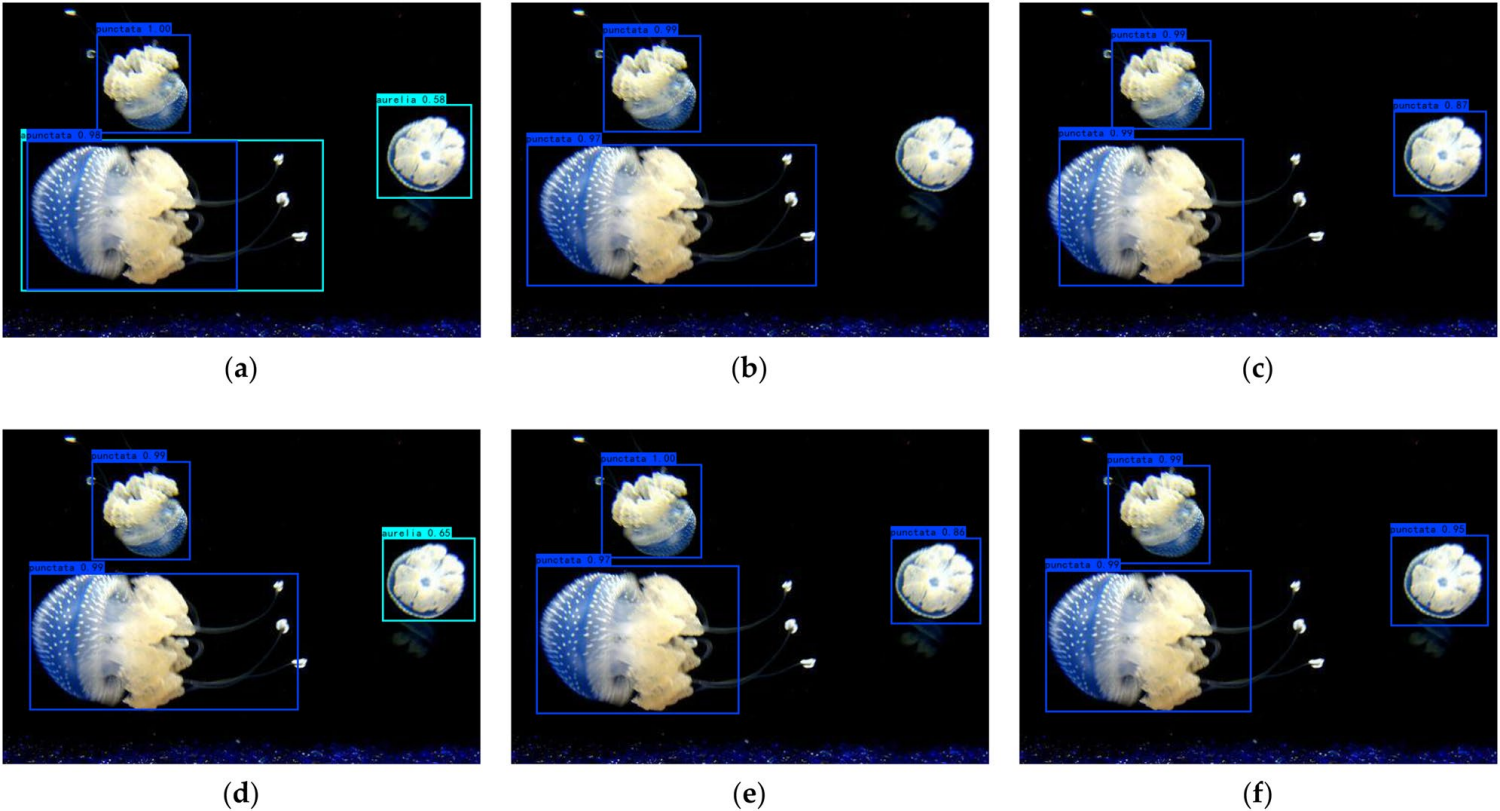
Results of ablation experiment for *P. punctata* jellyfish. (**a**) YOLOv4-tiny; (**b**) improved network structure; (**c**) improved network structure and mosaic enhancement; (**d**) improved network structure and cosine annealing learning rate; (**e**) improved network structure and label smoothing; (**f**) ours.
  Figure 10a illustrates that the YOLOv4-tiny algorithm mistakenly detects the right jellyfish as A. aurita and identifies the jellyfish in the left corner as both A. aurita and P. punctata. In Fig. 10b, the improved network correctly detects two *P. punctata* but overlooks the right jellyfish. Figures 10c,e,f display three evident *P. punctata* correctly identified by all six algorithms. However, the smaller and farther P. punctata are not recognized by any of the algorithms. Considering the confidence level, the comprehensively improved algorithm achieves confidence levels of 0.99, 0.99, and 0.95, respectively, with more accurate box positioning compared to other methods. Hence, the improved algorithm exhibits the best detection performance.
  Figure 11 shows the jellyfish video image captured in the field. The FPS value is displayed in the upper left corner, while the name and number above the bounding box indicate the accuracy of jellyfish species identification. The average FPS of the entire video is approximately 20, which is due to the large image resolution of 2448 * 2018.

**Figure 11:**
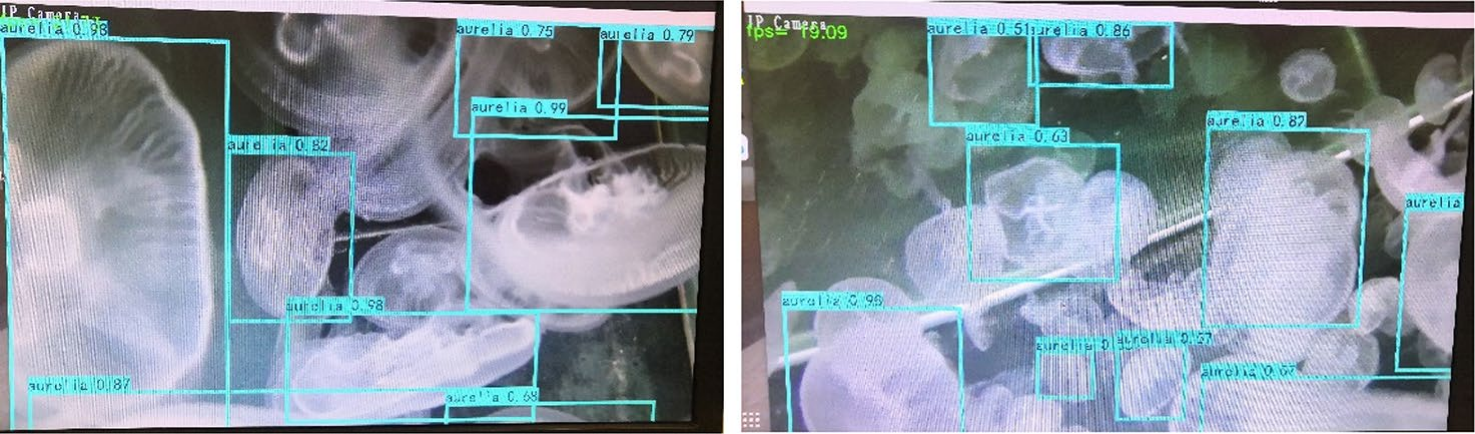
Video detection results of *A. aurita* jellyfish by comprehensively improved algorithm.
  Based on the analysis of experimental data, visualization effects, and performance metrics such as mAP, FPS, precision, recall, and F1-score, it is evident that the improved algorithm proposed in this paper outperforms the other algorithms. The improved algorithm achieves the best results in terms of mAP, FPS, precision, recall, and F1-score, indicating its superior detection capabilities. The experimental analysis also demonstrates that the improved algorithm produces the best detection effects for jellyfish examples.
  The high FPS value indicates the algorithm's ability to perform rapid detection, which is crucial for real-time applications. The high F1-score suggests that the network structure is stable, and the algorithm achieves a balanced performance in terms of precision and recall.
  Overall, the comprehensively improved algorithm presented in this paper enhances the accuracy of jellyfish detection while ensuring fast and efficient identification. It meets the requirements for rapid and accurate identification of jellyfish.

## Conclusions

This study addresses the demand for jellyfish detection by taking several important steps. Firstly, a new dataset containing a large number of images from seven jellyfish species is established, including both publicly available data and data collected in the laboratory. This dataset serves as a valuable resource for further research and development in the field of jellyfish detection. Next, to improve the quality of underwater images and enhance jellyfish detection, this paper proposes a MSRCR underwater image enhancement algorithm with fusion, and demonstrates the effectiveness and superiority of the proposed method through various objective image evaluation parameters. Futhermore, an improved YOLOv4-tiny jellyfish detection algorithm is proposed. This algorithm combines mosaic data augmentation, cosine annealing, and label smoothing methods for weight training, and incorporates CBAM modules to improve feature extraction capabilities, achieving both accuracy and real-time performance in jellyfish detection. Multiple evaluation results from YOLOv4 series ablation experiments and YOLO series comparative experiments demonstrate the superiority and practicality of the proposed algorithm, meeting the requirements for real-time and accurate detection of jellyfish.

While the proposed algorithm shows promising results, there are still challenges to overcome. These include slow processing speed for high-resolution videos, difficulties in handling multiple overlapping jellyfish scenes, and potential missed detections. Future research efforts will focus on improving the algorithm's ability to handle jellyfish overlap and increasing the processing speed for high-resolution images.

Overall, this study provides a template for jellyfish detection, and our proposed algorithm demonstrates good robustness and detection performance, with certain application and reference value in practical engineering detection. It highlights the research potential of the YOLO-tiny series method in jellyfish detection and sets the stage for future advancements in the field.

## Data Availability

One dataset is available in [Martin-Abadal, M. Jellyfish Object Detection] repository, https://github.com/srv/jf_object_detection; An additional portion of the dataset analyzed during the current study is available from the corresponding authors upon reasonable request.
